# Geographic origin shapes the adaptive divergences of *Rotaria rotatoria* (Rotifera, Bdelloidea) to thermal stress: Insights from ecology and transcriptomics

**DOI:** 10.1002/ece3.11307

**Published:** 2024-04-24

**Authors:** Meng Li, Fan Gao, Lingyun Zhu, Jianan Li, Jinjin Xiang, Yilong Xi, Xianling Xiang

**Affiliations:** ^1^ School of Ecology and Environment Anhui Normal University Wuhu Anhui China; ^2^ Collaborative Innovation Center of Recovery and Reconstruction of Degraded Ecosystem in Wanjiang Basin Co‐founded by Anhui Province and Ministry of Education Anhui Normal University Wuhu Anhui China

**Keywords:** genetic differences, geographic origin, local adaptation, zooplankton

## Abstract

Global warming has raised concerns regarding the potential impact on aquatic biosafety and health. To illuminate the adaptive mechanisms of bdelloid rotifers in response to global warming, the ecological and transcriptomic characteristics of two strains (HX and ZJ) of *Rotaria rotatoria* were investigated at 25°C and 35°C. Our results showed an obvious genetic divergence between the two geographic populations. Thermal stress significantly reduced the average lifespan of *R. rotatoria* in both strains, but increased the offspring production in the ZJ strain. Furthermore, the expression levels of genes *Hsp70* were significantly upregulated in the HX strain, while *GSTo1* and *Cu/Zn‐SOD* were on the contrary. In the ZJ strain, the expression levels of genes *Hsp70*, *CAT2*, and *GSTo1* were upregulated under thermal stress. Conversely, a significant decrease in the expression level of the *Mn‐SOD* gene was observed in the ZJ strain under thermal stress. Transcriptomic profiling analysis revealed a total of 105 and 5288 differentially expressed genes (DEGs) in the HX and ZJ strains under thermal stress, respectively. The PCA results showed clear differences in gene expression pattern between HX and ZJ strains under thermal stress. Interestingly, compared to the HX strain, numerous downregulated DEGs in the ZJ strain were enriched into pathways related to metabolism under thermal stress, suggesting that rotifers from the ZJ strain prioritize resource allocation to reproduction by suppressing costly metabolic processes. This finding is consistent with the life table results. This study provides new insights into the adaptive evolution of aquatic animals in the context of global climate change.

## Graphical abstract

Under global warming, all organisms are inevitably exposed to temperature stress. To gain a comprehensive understanding of how bdelloid rotifers respond to thermal stress, we investigated both the ecological and molecular responses of stress‐tolerant *Rotaria rotatoria*, and explored whether rotifers with different geographic origins exhibit differential responses to thermal stress.

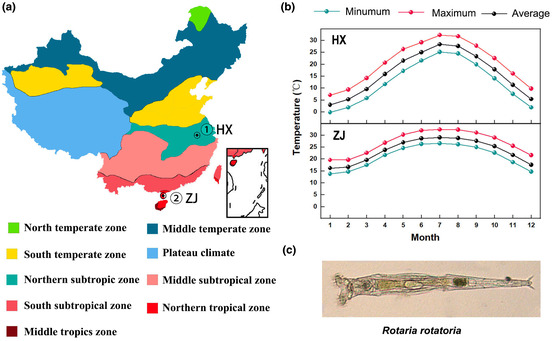

## 1 INTRODUCTION

Climate changes, such as heat waves and rising temperatures, are increasingly affecting aquatic ecosystems on a global scale (Parmesan, 2006; Yanik & Aslan, 2018). For aquatic zooplankton, temperature serves as a critical environmental factor, exerting profound influences on their physiological performances, spatial and temporal distribution, and habitat colonization (Gabaldón et al., 2017; Li et al., 2019; Rutherford et al., 1999; Zhang et al., 2018). These influences may be attributed to their poor self‐regulation of homeostasis under thermal stress and limited capacity to escape from the thermal environment (Boukadida et al., 2022; Dam & Baumann, 2017; Heinle, 1969). Consequently, understanding the thermal tolerance of aquatic organisms is crucial for both species distribution and population dynamics.

Understanding how rising temperatures affect the performance of individuals is essential to comprehending the natural population's responses to global warming, as a population's prosperity relies on the total of surviving individuals and newborns. The ecological response of individual zooplankton to thermal stress has been extensively investigated by using standard life table technology. This is typically manifested in increased resting egg production (Nhinh et al., 2020), reduced body size (Walczyńska et al., 2017), reduced generation time (Gillooly, 2000), accelerated respiration and heart rate (Santoso et al., 2020). Moreover, a common phenomenon associated with the response of physiology to thermal stress is the changes in the immune system. For example, Dölling et al. (2016) observed that thermal stress increased the activity of chymotrypsin, while slightly decreasing trypsin activity. Yang et al. (2013) found that the upregulation of *CuZn‐SOD* gene expression in a certain range plays an important role in maintaining the physiological equilibrium of *Brachionus calyciflorus*. In addition, stress proteins such as heat shock proteins and antioxidant enzymes also play an important role in protecting zooplankton species from thermal stress (Roberts et al., 2010), which has been extensively studied in many aquatic organisms (González et al., 2016; Xiang et al., 2017). Although numerous studies have examined the ecological and physiological responses of aquatic organisms to temperature stress, there is still a dearth of research on transcriptomics.

Temperatures of a habitat vary spatially and temporally and such variation can serve as a significant selective force that leads to the genetic divergence of local populations (Angilletta, 2009). Generally, temperate ectothermic species, inhabiting mid‐latitudes with clear seasonal differences, have wider thermal performance ranges and are more cold‐tolerant and less heat‐tolerant compared to tropical species (Pörtner & Peck, 2010; Spicer et al., 2019). Moreover, populations of an allopatric species with wide geographic distribution also have their ecological and physiological traits to adapt to ambient temperature fluctuation. For example, rotifers, inhabiting different geographic regions, undergo long‐term natural selection resulting in differences in their morphological and ecological traits such as life‐history traits, population growth, body size, and egg sizes (Awaïss & Kestemont, 1992; Hagiwara et al., 1995; Xi et al., 2002). Importantly, similar species might have evolved species‐specific genetic traits to temperature (Papakostas et al., 2013). Therefore, knowledge of genetic differences of aquatic organisms with different geographic origins is essential to understand how species have adapted to their environment and how they may respond to climate change.

Bdelloid rotifers are a class of unusual microscopic invertebrates in freshwater ecosystems because of two characteristics. Firstly, they are known to reproduce via mitotic parthenogenesis, rendering them an ideal model for investigating asexual reproduction in eukaryotic research (Flot et al., 2013; Simion et al., 2021). Secondly, bdelloid rotifers exhibit remarkable tolerance to harsh environments such as ionizing radiation (Gladyshev & Meselson, 2008), drought (Nowell et al., 2018; Ricci et al., 2003), and heavy ions (Jönsson & Wojcik, 2017). As such, they serve as an excellent model for exploring the stress tolerance of organisms in unfavorable environments. In the context of global warming, all organisms are inevitably exposed to temperature stress. However, most studies have primarily focused on organisms that are susceptible to environmental stress, neglecting the examination of rotifers that exhibit resistance to such conditions. Consequently, we hypothesize that stress‐tolerant rotifers may possess distinct response characteristics and mechanisms when confronted with thermal stress. In order to gain a comprehensive understanding of how bdelloid rotifers respond to thermal stress, this study investigates both the ecological and molecular responses of stress‐tolerant *Rotaria rotatoria*. Additionally, we also explore whether rotifers with different geographic origins exhibit differential responses to thermal stress.

## 2 MATERIALS AND METHODS

### 2.1 Experimental organism

#### 2.1.1 Sample collection and culture

**FIGURE 1:**
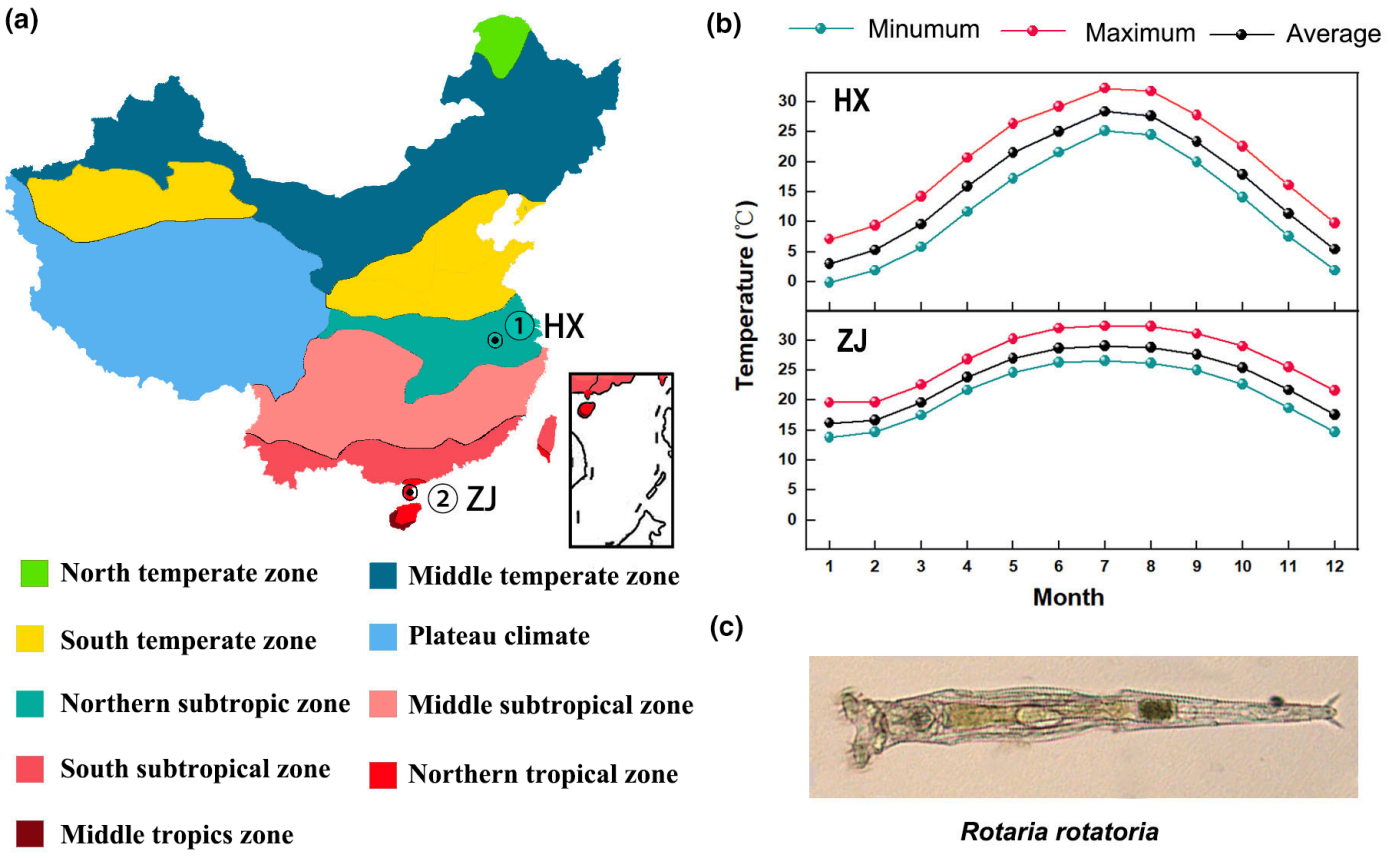
Information of sampling sites and experimental organism. (a) Geographic location; (b) temperature fluctuation of sampling sites (obtained from China Meteorological Administration, http://www.cma.gov.cn/); (c) photograph of *Rotaria rotatoria*.

The *R. rotatoria* individuals were sampled from two geographic areas: Hexian (HX) and Zhanjiang (ZJ). The HX strain rotifers were obtained from a pond near Jing village in Hexian (31°73′ N, 118°33′ E) in September 2013, with the water temperature documented at 22.61°C. Conversely, the ZJ strain rotifers were collected from a pond at Guangdong Ocean University in Zhanjiang (21°15′ N, 110°30′ E) in August 2013, where the water temperature was noted at 24.83°C. HX sits on the northern subtropical zone of China with four distinct seasons, characterized by cold winters and muggy summers, while ZJ is situated in the northern boundary of China's tropical zone, and is known for its humid subtropical climate with mild winters and sultry summers (Figure 1a). HX has an average annual temperature of 17.9°C, exhibiting an obvious annual temperature range, whereas ZJ has an average annual temperature of 24.4°C, displaying a small annual temperature fluctuation (Figure 1b).

After the collection, individual rotifers were clonally cultured in a sterile EPA medium (Peltier & Weber, 1985) under natural light (≈1300 Lux) at 25 ± 1°C with green algae *Tetradesmus obliquus* at a density of 1.0 × 10^6^ cells/mL. The algae were semi‐continuously cultured in HB‐4 medium with illumination of 3000 Lux at 28°C (Andersen, 2005). To ensure optimal cultural conditions, the EPA medium was renewed daily, and the algal food was provided every 24 h during cultivation. As each rotifer clone grew steadily and produced offspring successfully (≥10 ind.), they were identified as the *R. rotatoria* species using the microscopic discrimination of morphological characters (Figure 1c) and mitochondrial COI gene marker following the method described in Xiang et al. (2023). Finally, a total of 33 and 20 individuals were successfully collected from HX strains and ZJ strains, respectively. For mitochondrial COI gene, all sequences have been deposited in GenBank (accession numbers: KT438170–KT438202 for HX strain and OR648093–OR648112 for ZJ strain). To reveal the genetic differences between the two strains of rotifers, a phylogenetic tree was constructed by using these sequences. For conducting phylogenetic reconstruction, all sequences were analyzed using the maximum likelihood method in MrBayes software, integrated in PhyloSuite version 1.2.2 (Zhang et al., 2020). The ModelFinder tool was utilized to determine the optimal model for constructing MrBayes tree (Kalyaanamoorthy et al., 2017). Moreover, population genetic statistics within species, including nucleotide diversity (Pi), average number of nucleotide differences (K), and average number of segregating sites (S) were calculated using DNASP v6 (Librado & Rozas, 2009).

#### 2.1.2 Mass culture and thermal treatment of rotifers

Two clones HX8 and ZJ18 were randomly selected as the representatives of two strains for ecological experiments, gene expression and RNA‐seq analysis. They were mass cultured using a sterile EPA medium at 25°C until achieving a prosperous population (>1.0 × 10^5^ ind.). To provide optimal conditions for cultivation, green algae with a density of 1.0 × 10^6^ cells/mL were used as the food source. The EPA medium was renewed daily to ensure optimal growth conditions, and the algae were replenished every 24 h to avoid food shortages. In order to ensure that the rotifers were not restricted by their population density, the density of the rotifers was controlled at 50–100 ind./mL.

Before thermal treatment, the EPA medium used in the experiment was placed in the bioclimatic chamber at the experimental temperature of 35°C for 24 h to ensure that rotifers were not influenced by the supply of thermal stress. Subsequently, a mass culture of rotifers, consisting of 1.0 × 10^5^ individuals, were transferred into EPA medium with water temperatures of 35°C, and then placed in a bioclimatic chamber at 35°C for 24 h. During thermal treatment period, rotifers were starved to prevent any potential intracorporal food contamination. After that, the rotifers were collected using a process of filtration and centrifugation (6000 rpm, 5 min) at the experimental temperature. Subsequently, the rotifers were deposited into 1.5 mL of freezing tubes and then were immediately transferred to liquid nitrogen for flash freezing. All samples were stored at −80°C until they were needed for subsequent RNA‐seq and RT‐qPCR experiments.

### 2.2 Ecological experiment

We used newborn rotifers (≤6 h old) from two clones derived from stock cultures for the ecological experiment. They were each placed individually into rearing containers containing 0.5 mL of sterile EPA medium with 1.0 × 10^6^ cells/mL of algae. The rearing containers were placed into a bioclimatic chamber with natural illumination at 25°C (control) and 35°C (treatment: thermal stress). Each treatment was replicated eight times for each strain. The living rotifers and newborn neonates were counted every 12 h, and then neonates and dead animals were removed. The EPA medium and fresh food were replaced every 24 h until the whole cohort died. Based on the recorded data, the average lifespan and the offspring production in different temperatures were calculated.

### 2.3 Quantitative analysis of gene expression in *R. rotatoria* under thermal stress

To determine the expression level of thermal‐related genes, including *hsp70*, *CAT2*, *Cu/Zn‐SOD*, *Mn‐SOD*, and *GSTo1* in *R. rotatoria* under thermal stress, the RT‐qPCR experiment was performed using the Bio‐Rad CFX96 Touch Real‐Time PCR Detection System (Bio‐Rad Laboratories, CA). The total RNA of *R. rotatoria* (approximately 3000 individuals), from each treatment, were isolated by using Trizol (Invitrogen, USA) following the protocol. The quality of RNA was verified through electrophoresis on a 1.2% agarose gel and by measuring the absorbance ratio at *λ*260/*λ*280 nm. All isolated RNA samples were stored at −80°C until they were needed for subsequent experiments. DNA library was constructed by utilizing the PrimeScriptTMRT Reagent Kit (TaKaRa, Japan).18sRNA gene (GenBank: JX494744) was used as the reference gene since its expression levels did not show significant changes within the different treatments. Specific primers utilized are available in Table S1. The reaction protocol entailed the following: incubation at 95°C for 30 s; 45 cycles at 95°C for 5 s, 59°C for 15 s, and 72°C for 20 s, followed by 72°C for 7 min. Three biological replicates for each sample were detected, and we presented all data in terms of relative expression using the 2^−ΔΔCT^ method to calculate the relative mRNA expression (Livak & Schmittgen, 2001).

### 2.4 Transcriptomics analysis in *R. rotatoria* under thermal stress

Total RNA was extracted from rotifer population (approximately 1 × 10^5^ individuals) with TRIzol reagent (Invitrogen, CA, USA), following the manufacturer's protocol. The concentration and integrity of the RNA were determined using Bioanalyzer 2100 and RNA 6000 Nano LabChip Kit (Agilent, CA, USA) with a RIN (RNA integrity number) ≥ 7.0. Sequencing libraries were obtained following the handbook for the NEBNext Ultra RNA Library Prep Kit for Illumina (NEB, USA). Index codes were added to attribute sequences to each sample. Poly(A) RNA was purified from 5 μg of the total RNA sample using poly‐T oligo‐attached magnetic beads and two rounds of purification. The mRNA was fragmented into small pieces using divalent cations under elevated temperature conditions. The fragmented RNA was then reverse‐transcribed to construct the final cDNA library. The protocol for the mRNASeq sample preparation kit (Illumina, San Diego, USA) was followed, with an average insert size of 300 bp (±50 bp) for the paired‐end libraries. Paired‐end sequencing was performed using an Illumina Novaseq™ 6000 at LC Sciences (USA), in adherence to the vendor's recommended protocol.

The reads containing adapter contamination, low‐quality bases, or undetermined bases were filtered using Cutadapt (Martin, 2011) and in‐house Perl scripts (Gong et al., 2015). Subsequently, FastQC (http://www.bioinformatics.babraham.ac.uk/projects/fastqc/) was employed to verify the quality of sequences, including the Q20, Q30, and GC‐content of the clean data that was considered for downstream analysis. De novo assembly of the transcriptome was carried out using Trinity 2.4.0 (Grabherr et al., 2011). Each transcript cluster was loosely referred to as a ‘gene’, with its longest transcript selected as the ‘gene’ sequence (aka unigene). Expression levels of unigenes were computed via TPM (Mortazavi et al., 2008), using Salmon (Patro et al., 2017). The choice of DEGs was determined using the R package, edgeR (Robinson et al., 2010) and based on the following criteria: an absolute value of log_2_(fold change) greater than or equal to 1 and a *p*‐value less than .05. Following the identification of DEGs, a functional enrichment analysis of KEGG pathway was carried out. An enrichment analysis via a hypergeometric test was performed on pathways. Those with *p*‐values ≤.05 were considered to have a significant enrichment of DEGs.

### 2.5 Statistical analysis

The statistical analysis was conducted using IBM SPSS 25.0. The data were assessed for normality and homogeneity of variance using the Shapiro–Wilk test and Levene's test, respectively. If necessary, the data were log‐transformed. Significant differences between the control and treatment groups, as well as between the HX and ZJ strains, were determined using independent samples *t*‐tests (two‐tailed). Two‐way ANOVA was used to analyze the effects of strain, temperature, and their interactions on the average lifespan, offspring production, and relative expression levels of genes. A *p*‐value of less than .05 was considered statistically significant. Furthermore, Arraytrack software was used to perform PCA and elucidate gene expression patterns between the two strains of rotifers under different temperature conditions (Fang et al., 2017).

## 3 RESULTS

### 3.1 Phylogenetic analysis of two rotifer strains

**FIGURE 2:**
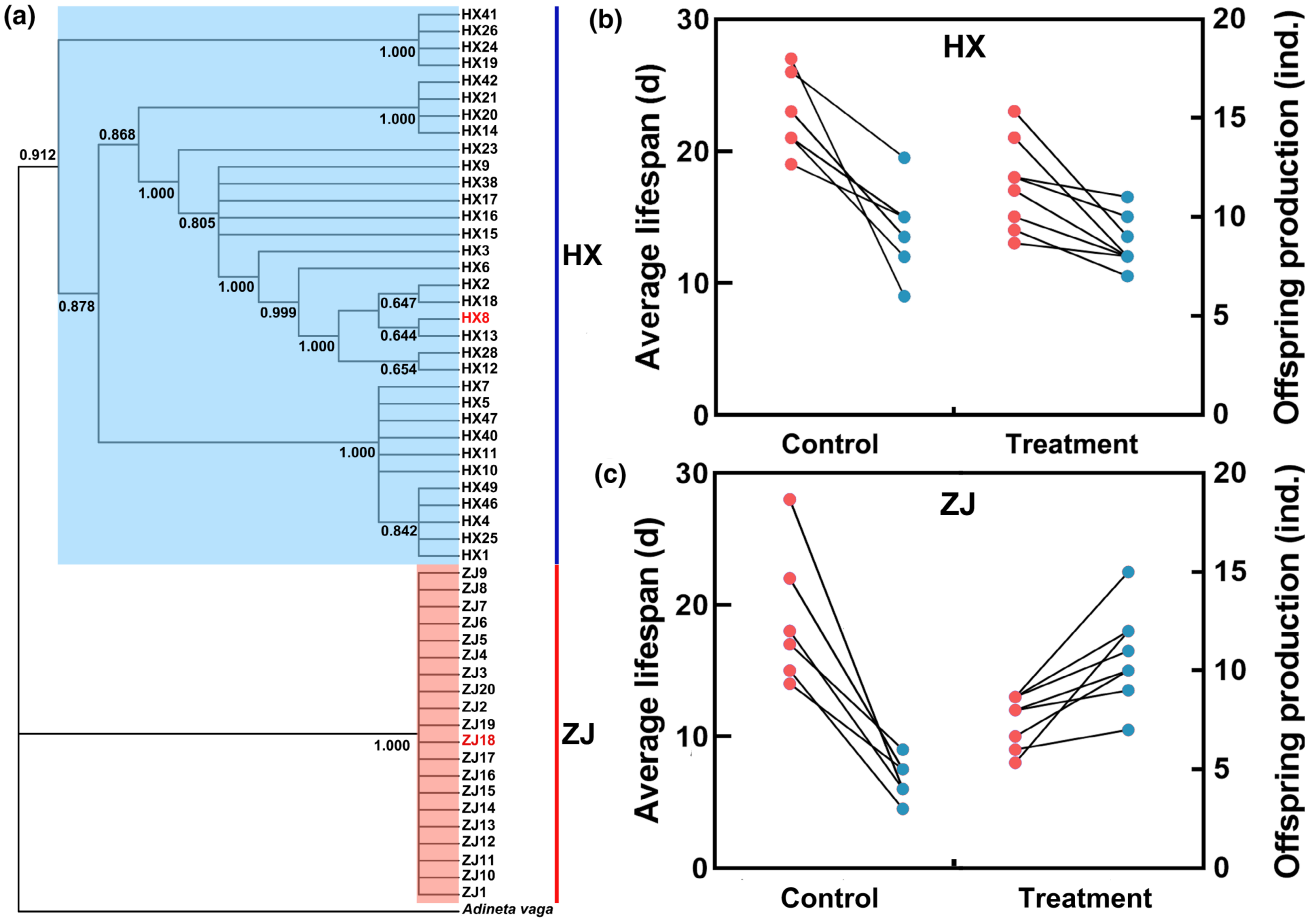
Phylogenetic relationships and life‐history characteristic of *Rotaria rotatoria*. (a) Phylogenetic tree; (b). Average lifespan and offspring production in the HX strain; (c) Average lifespan and offspring production in the ZJ strain. The red and the blue circles represent average lifespan and offspring production, respectively.

Using Bayesian phylogenetic analysis, we successfully reconstructed the phylogenetic tree of *R. rotatoria* (Figure 2a). All individuals analyzed were divided into two distinct groups. Group 1 (posterior probability = 0.912) consisted of 33 individuals exclusively from HX in humid subtropical climate. In contrast, Group 2 (posterior probability = 1.000) comprised 20 individuals from ZJ in tropical monsoon climate, forming independent clades based on regional climates. The results showed a high genetic divergence between the two geographic populations. In addition, greater genetic variation was also observed in HX than in ZJ, with higher nucleotide diversity, average number of nucleotide differences, and average number of segregating sites (Table 1).

**TABLE 1:** Genetic diversity summary statistics for the two rotifer strains.

Samples	Individuals	Nucleotide diversity (Pi)	Average number of nucleotide differences (*K*)	Average number of segregating sites (*S*)
HX	33	0.07835	43.87500	111
ZJ	20	0.00429	2.40000	24

### 3.2 Growth and reproduction performance

The results showed that the average lifespan of HX and ZJ strains was significantly decreased by 23.2% and 45.1%, respectively, under thermal stress (HX: *F* = 0.329, df = 14, *p* = .004; ZJ: *F* = 8.734, df = 14, *p* = .000, Figure 2b). Although no significant difference in offspring production was observed in the HX strain (*F* = 0.411, df = 14, *p* = .388), thermal stress significantly increased the offspring production of the ZJ strain by up to 2.4‐fold (*F* = 3.394, df = 14, *p =* .000, Figure 2c). Additionally, we observed that the average lifespan of HX strain was significantly higher than that of the ZJ strain under thermal stress (*F =* 1.564, df = 14, *p* = .001). Meanwhile, the offspring production of the HX strain was significantly higher than that of the ZJ strain at 25°C (*F* = 1.535, df = 14, *p* = .000); however, there was no significant difference in offspring production between the two strains at 35°C (*F* = 1.575, df = 14, *p* = .230). The two‐way ANOVA analysis showed significant effects of strain, temperature, and their interaction on average lifespan and offspring production, with the exception of the interaction between strain and temperature on average lifespan (Table 2).

**TABLE 2:** Results for average lifespan and offspring production with two‐way ANOVA.

	Parameter	df	SS	MS	*F*	*p*
Average lifespan	Strain (S)	1	420.500	420.500	31.886	.000
	Temperature (T)	1	136.125	136.125	10.322	.003
	S × T	1	32.000	32.000	2.427	.131
Offspring production	Strain (S)	1	60.500	60.500	19.871	.000
	Temperature (T)	1	15.125	15.125	4.968	.034
	S × T	1	98.000	98.000	32.188	.000

### 3.3 Quantitative analysis of thermal‐related genes

**FIGURE 3:**
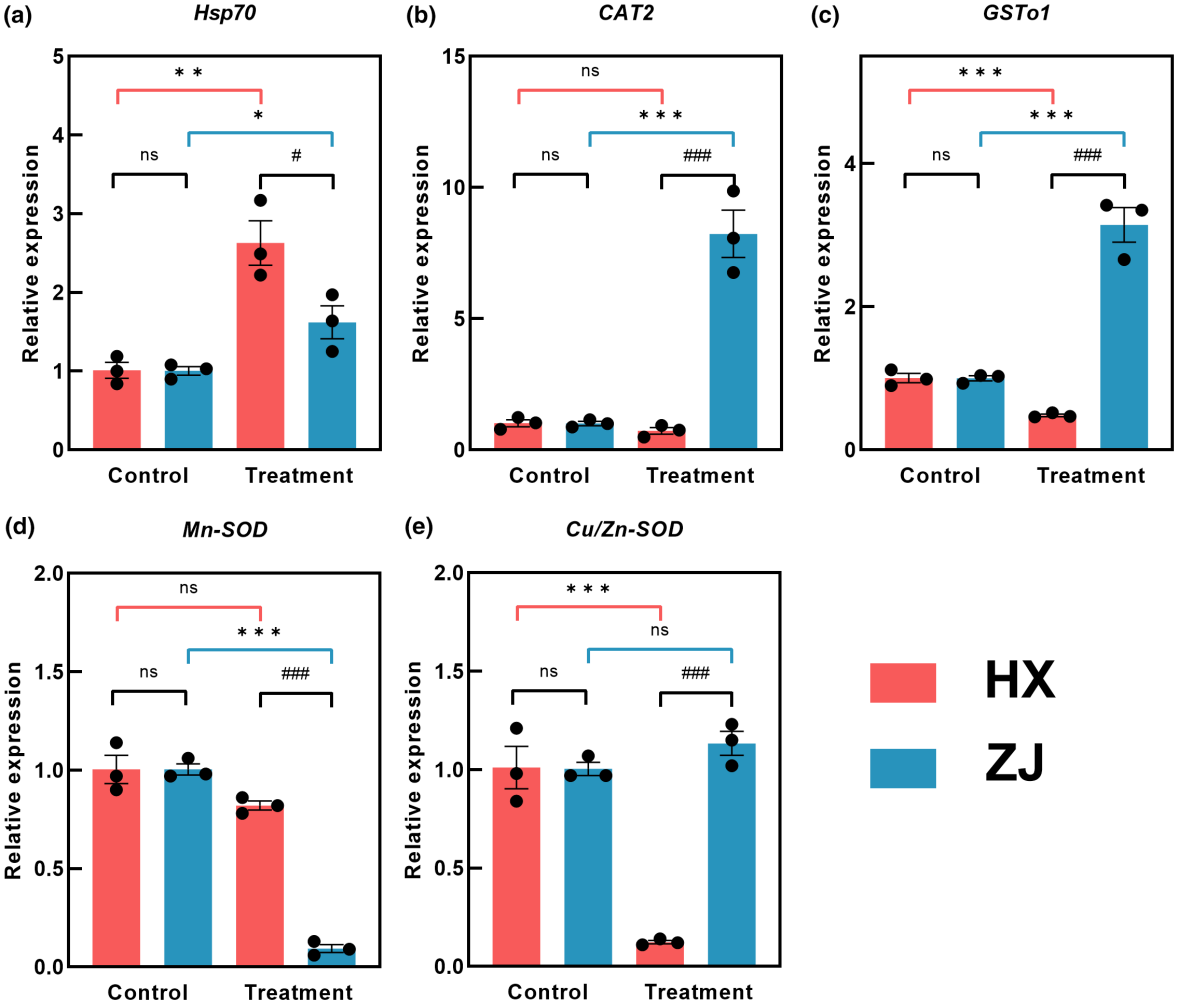
Relative expression of *Hsp70*, *CAT2*, *GSTo1*, *Mn‐SOD*, and *Cu/Zn‐SOD* genes in *Rotaria rotatoria* after 24 h of thermal stress (Mean ± SE). The asterisks *, **, and *** indicate statistical differences between HX and ZJ strains with significant levels of .05, .01, and .001, respectively; The notation #, ##, and ### indicate significant differences between control and treatment with significant levels of .05, .01, and .001, respectively.

The RT‐qPCR analysis revealed regulation patterns of genes related to thermal stress in both the HX and ZJ strains (Figure 3). Specifically, in the HX strain, only one gene *Hsp70* was significantly upregulated following exposure to thermal stress (*F* = 3.390, df = 4, *p* = .006). However, the genes *GSTo1* and *Cu/Zn‐SOD* exhibited significant downregulation compared to the control under thermal stress in the HX strain (*GSTo1*: *F* = 2.589, df = 4, *p* = .001; *Cu/Zn‐SOD*: *F* = 5.411, df = 4, *p* = .001). In the ZJ strain, thermal stress resulted in a significant upregulation in relative expression levels of genes *Hsp70*, *CAT2*, and *GSTo1* (*Hsp70*: *F* = 2.362, df = 4, *p* = .045; *CAT2*: *F* = 4.518, df = 4, *p* = .001; *GSTo1*: *F* = 10.783, df = 4, *p* = .001). Moreover, a significant downregulation of 10.802‐fold was observed in the *Mn‐SOD* gene expression in the ZJ strain under thermal stress (*F* = 0.847, df = 4, *p* = .000). Notably, no significant differences were found in the expression levels of the five genes between the HX and ZJ strains at the control temperature of 25°C (*Hsp70*: *F* = 0.738, df = 4, *p* = .956; *CAT2*: *F* = 0.537, df = 4, *p* = .952; *GSTo1*: *F* = 0.808, df = 4, *p* = .966; *Mn‐SOD*: *F* = 2.776, df = 4, *p* = 1.000; *Cu/Zn‐SOD*: *F* = 2.755, df = 4, *p* = .956). However, the ZJ strains exhibited a significant upregulation in the expression levels of *CAT2*, *GSTo1*, and *Cu/Zn‐SOD* genes, with folds of 11.35, 6.49, 1.68, and 9.19 compared to the HX strain at the treatment temperature of 35°C (*CAT2*: *F* = 3.949, df = 4, *p* = .001; *GSTo1*: *F* = 12.771, df = 4, *p* = .000; *Cu/Zn‐SOD*: *F* = 4.583, df = 4, *p* = .000). In contrast, the expression levels of the *Hsp70* and *Mn‐SOD* genes were found to be significantly downregulated in the ZJ strains, with folds of 1.62 and 8.85, respectively, compared to the HX strain under thermal stress of 35°C (*Hsp70*: *F* = 0.492, df = 4, *p =* .046; *Mn‐SOD*: *F* = 0.017, df = 4, *p* = .000). The results of the two‐way ANOVA analysis indicated that the relative expression levels of *Hsp70*, *CAT2*, *GSTo1*, *Mn‐SOD*, and *Cu/Zn‐SOD* were significantly affected by strain, temperature, and their interaction (Table 3).

**TABLE 3:** Results for heat stress‐related gene expression with two‐way ANOVA.

Genes	Parameter	df	SS	MS	*F*	*p*
*Hsp70*	Strain (S)	1	0.770	0.770	7.534	.025
	Temperature (T)	1	3.741	3.741	36.597	.000
	S × T	1	0.750	0.750	7.337	.027
*CAT2*	Strain (S)	1	42.188	42.188	65.927	.000
	Temperature (T)	1	36.053	36.053	56.341	.000
	S × T	1	42.413	42.413	66.279	.000
*GSTo1*	Strain (S)	1	5.293	5.293	109.481	.000
	Temperature (T)	1	1.976	1.976	40.877	.000
	S × T	1	5.320	5.320	110.031	.000
*Mn‐SOD*	Strain (S)	1	0.396	0.396	77.275	.000
	Temperature (T)	1	0.897	0.897	174.933	.000
	S × T	1	0.396	0.396	77.275	.000
*Cu/Zn‐SOD*	Strain (S)	1	0.755	0.755	60.765	.000
	Temperature (T)	1	0.429	0.429	34.560	.000
	S × T	1	0.755	0.755	62.391	.000

### 3.4 Exploratory analysis of RNA‐seq

#### 3.4.1 Global effects of thermal stress on transcriptome

**FIGURE 4:**
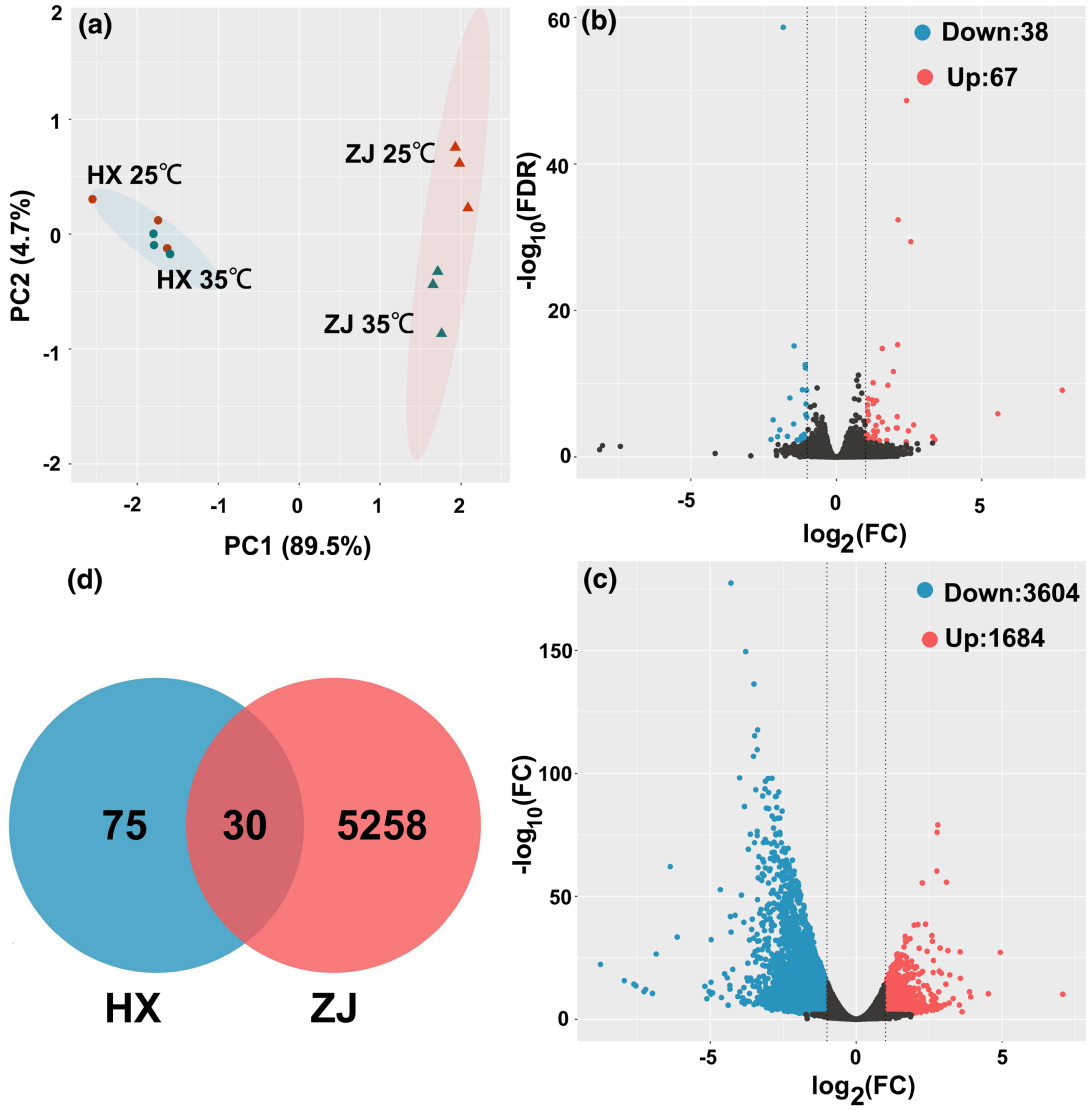
Transcriptome data analysis. (a) PCA analysis of gene expression profiles. (b) DEGs in HX strain. (c) DEGs in ZJ strain. (d) Shared and unique genes numbers between HX and ZJ.

In order to elucidate the molecular mechanisms involved in thermal stress, we continued our investigation by comparing transcriptomes obtained from two strains of rotifers. Each sample yielded an average of 43.28 million reads. After filtering, all samples yielded valid reads with a rate ranging from 89.58% to 95.08%. For each sample, more than 97% of bases achieved a score of Q30 or higher, indicating the potential usefulness of the sequencing results for subsequent analysis (Table S2). To determine the global effects of thermal stress on gene transcription, principal component analysis (PCA) was conducted on the relative expression levels of all genes (Figure 4a). The PCA results revealed clear differences in gene expression between HX and ZJ strains under thermal stress, indicating great differences in adaptive mechanisms in the two strains.

In addition, a total of 105 and 5288 DEGs are in HX and ZJ strains, respectively. Of these, 67 genes were upregulated and 38 genes downregulated in HX strains (Figure 4b, Table S3), while 1684 and 3604 genes were upregulated and downregulated in ZJ strains, respectively (Figure 4c, Table S4). In terms of the number of DEGs, the ZJ strain showed a significantly greater number of DEGs than the HX strain. Only 30 DEGs were shared between the two strains (Figure 4d). Thus, it can be inferred that the difference in adaptive mechanisms in *R. rotatoria*, caused by the geographic origins, cannot be ignored under thermal stress.

#### 3.4.2 KEGG functional classification of the DEGs

**FIGURE 5:**
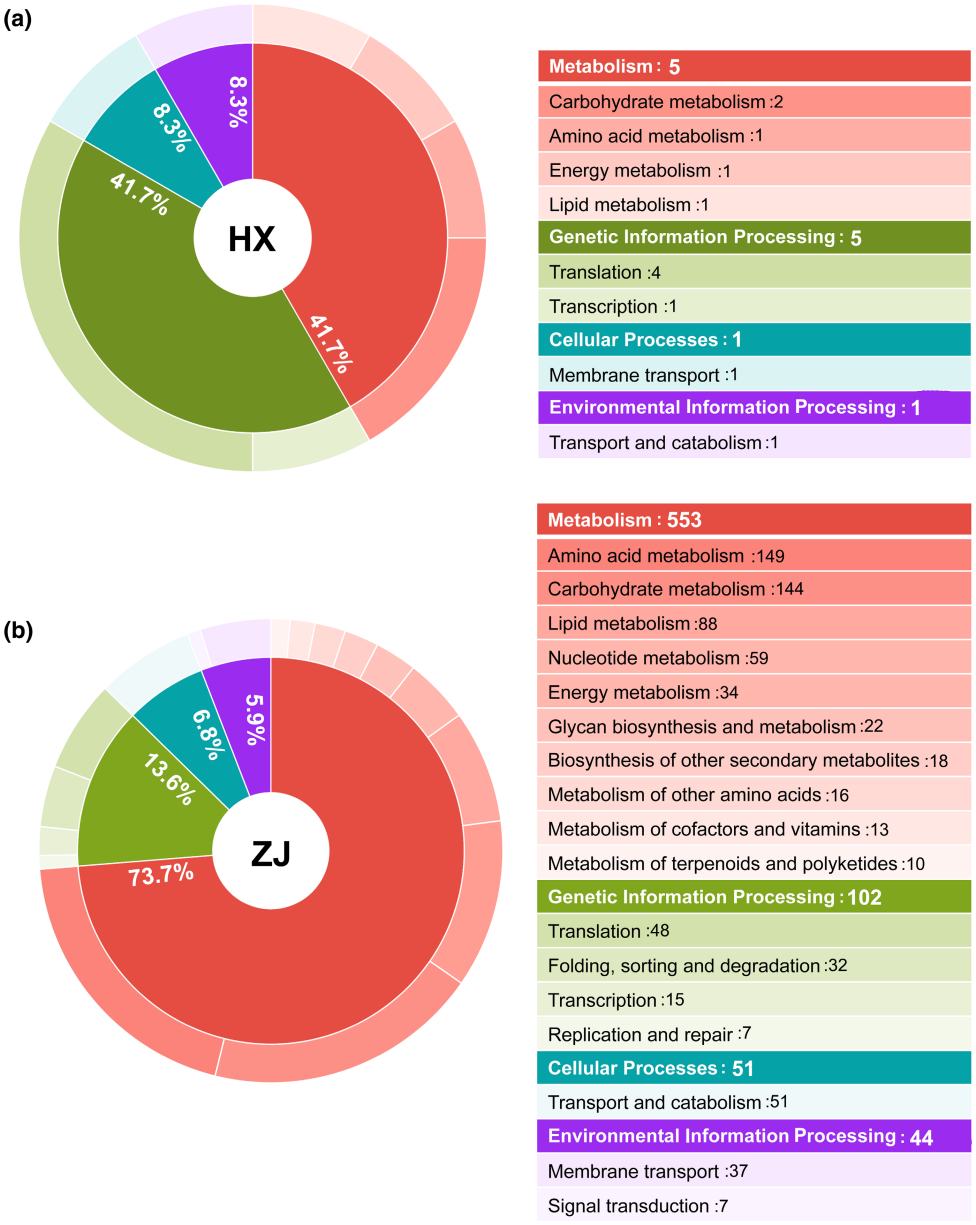
Distribution of KEGG pathways in main and secondary biological categories. (a) *Rotaria rotatoria* collected from HX; (b) *R. rotatoria* collected from ZJ.

The KEGG enrichment pathway analysis sheds light on how animal metabolic pathways respond to thermal stress, as shown in Figure 5. Both HX and ZJ strains, the enrichment results of DEGs were predominantly associated with metabolism, genetic information processing, cellular processes, and environmental information processing. Notably, metabolism emerged as a significant pathway that exhibited substantial enrichment, underscoring the profound impact of thermal stress on metabolic processes. However, a notable disparity was observed between the HX and ZJ strains in terms of the number of DEGs related to metabolism. Specifically, the HX strain exhibited enrichment of only five DEGs in metabolism‐related pathways, whereas a striking total of 553 DEGs were enriched in metabolism‐related pathways in the ZJ strain. These pathways primarily encompassed carbohydrate metabolism, amino acid metabolism, and lipid metabolism. In these pathways, 58.3% and 80.4% genes were downregulated in the HX and ZJ strains under thermal stress, respectively (Table S3).

## 4 DISCUSSION

### 4.1 Thermal adaptation in life‐history strategy

With the change of climate, the current rate of global warming is increasing. As metazoans, the thermal performance curves of zooplankton are narrower than those of their unicellular protist prey, making zooplankton more vulnerable to warming (Dam & Baumann, 2017). Ecologically, life‐history traits and their sensitivity to temperature changes played key roles in regulating the response of organisms to climate change, and organisms usually adjust their life‐history traits to adapt to environmental changes (Atkinson & Sibly, 1997; Stelzer, 2005). For example, the lifespan of short‐spined morph of *Keratella tropica* at 20°C was significantly higher than that at 25°C and 30°C, while the net reproductive rate was on the contrary (Ge et al., 2020), which were the responses of life‐history traits to the changes of temperature. And in this study, the huge magnitude of changes in the lifespan and reproduction occurred in ZJ strains, those were the number of offspring increasing and the lifespan decreasing, with the temperature increasing. It could be largely explained that the rotifer of ZJ had trade‐off strategies to increase the energy investment in reproduction and to decrease the energy in survival when they were subjected to thermal stress, which was similar to that of *Daphnia magna* (Adamczuk, 2020). However, there existed fewer changes in the lifespan and reproduction of HX strains in this study indicating that the sensitivity of life‐history traits to environmental changes in variable strains was different, which may be mainly explained by the local specificity of life history produced by the adaptation of various strains to different habitats (Bowman & Post, 2023; Stelzer, 2005). Furthermore, it is noteworthy that the HX strain displayed a higher offspring production capacity than the ZJ strain at 25°C. Conversely, at 35°C, the ZJ strain did not yield a significantly higher number of offspring than the HX strain. This suggests that the reproductive activity of HX strain rotifers, accustomed to regions with notable temperature fluctuations, appears to be less impacted by temperature fluctuations when compared to ZJ strain rotifers dwelling in areas with more stable temperature conditions. These findings revealed that rotifer species residing in varied climatic environments have developed species‐specific temperature response mechanisms aligned with their respective life strategies, which was similar to that of *Drosophila* species (Ito & Awasaki, 2022).

### 4.2 Thermal adaptation in antioxidant defense system

Extreme thermal profile changes could induce the unregular expression of heat shock protein genes, one of the conservative mechanisms belonging to evolution (Feder & Hofmann, 1999). In the present study, both two strains of rotifers significantly increased the expression levels of the *Hsp70* gene under thermal stress, especially in the HX strain, which indicated the rotifers strain from the region with more fluctuated temperature (i.e., HX) adapted to thermal stress better than that from the region with less fluctuated temperature (i.e., ZJ). In addition, antioxidant enzyme genes also made responses to thermal stress. Especially, significant abnormal regulations (fold changes > 3) of the expression levels in genes *CAT2*, *GSTo1*, and *Mn‐SOD* occurred only in the ZJ strain, which may be a result of accelerating metabolism under thermal stress (Wojewodzic et al., 2011), so the ROS could be eliminated to reduce oxidative damage (Kaneko et al., 2005). However, the significant upregulation of the *GSTo1* expression only occurred in the HX strain, which might increase the thermal tolerance of the HX strain (Lim et al., 2016). Overall, the HX strain coped with thermal stress better than the ZJ strain via fewer abnormal gene expressions.

### 4.3 Thermal adaptation in transcriptomic

In this study, although multiple genes associated with thermal stress were identified, our exploratory analysis of RNA‐seq mainly focused on the genes related to metabolism. In ectotherms, metabolism is inextricably related to environmental temperature, and this is usually reflected in the fact that when the temperature rises, the metabolic rate accelerates (Clarke & Fraser, 2004). Compared with HX strains, most metabolism‐related genes in ZJ strains were repressed and participated in the carbohydrate, amino acid, and lipids metabolism. Thus, thermal stress disorders the metabolic systems of *R. rotatoria*, which explains reduced average lifespan and increased offspring production in ZJ strains.

Amino acid, carbohydrate, and lipid metabolism are highly conserved processes that have a significant impact on various aspects of an organism's biology (Watts & Ristow, 2017). During digestion, lipids and carbohydrates are broken down into fatty acids and simple sugars, which are essential for the production of a wide range of metabolites necessary for development and survival (Watts et al., 2003). Generally, the expression levels of genes related to carbohydrate metabolism were upregulated after thermal exposure in many aquatic organisms, such as teleost fish *Gillichthys mirabilis* (Buckley et al., 2006) and Pacific oyster, *Crassostrea gigas* (Yang et al., 2017), which may be attributed to a need for rapid production of ATP under increasing temperatures. However, studies from rotifers reveal that heat‐tolerant *B. calyciflorus* has adapted to maintain its metabolism under high temperature, while the heat‐sensitive *B. fernandoi* apparently shuts down costly metabolic processes in order to allocate available resources to survival (Paraskevopoulou et al., 2020). In this study, only a limited number of genes related to lipid and carbohydrate metabolism showed differential expression under thermal stress in the HX strain. In contrast, in the ZJ strain, large numbers of genes involved in amino acid, lipid, and carbohydrate metabolism were downregulated under thermal stress. This suggests that HX strain of *R. rotatoria* has a strong tolerance to thermal stress, while those of the ZJ strain is more sensitive to it. Based on the downregulation genes in metabolic‐related pathways, it is obvious that *R. rotatoria* shuts down costly metabolic processes in order to allocate available resources to reproduction.

## 5 CONCLUSION

Through comparative analysis of bdelloid rotifers from Hexian and Zhanjiang, we found obvious genetic differences in the rotifer populations between the two regions, and they also exhibit different responses to temperature stress. The adaptability of species to thermal stress is related to the scale of temperature fluctuations in their habitats. Specially, individuals from regions with large‐scale temperature fluctuations displayed higher tolerances to thermal stress in terms of growth and reproduction than individuals from regions with small‐scale temperature fluctuations. This phenomenon would be associated with the difference in antioxidant defense system and metabolism of individuals with different geographic origins. While thermal stress induced regulation of heat‐related genes, including *Hsp70*, *CAT2*, *GSTo1*, *Mn‐SOD*, and *Cu/Zn‐SOD*, there are inconsistencies in gene expression levels and pattern among individuals with different geographic origins. Moreover, transcriptomic profiling analysis revealed that individuals inhabiting in region with small‐scale temperature fluctuations suppress costly metabolic processes to allocate available resources toward reproduction. This study provides new insights into adaptive evolution in aquatic animals and opened avenues for understanding adaptation strategies and mechanisms of aquatic animals under global warming.

## AUTHOR CONTRIBUTIONS

**Meng Li:** Conceptualization (supporting); data curation (lead); investigation (supporting); methodology (equal); writing – original draft (lead); writing – review and editing (supporting). **Fan Gao:** Data curation (supporting); methodology (supporting); writing – original draft (supporting). **Lingyun Zhu:** Data curation (supporting); investigation (supporting); writing – original draft (supporting). **Jianan Li:** Conceptualization (supporting); investigation (lead); methodology (supporting). **Jinjin Xiang:** Data curation (supporting); investigation (supporting). **Yilong Xi:** Conceptualization (supporting); methodology (supporting); writing – review and editing (supporting). **Xianling Xiang:** Conceptualization (lead); data curation (supporting); funding acquisition (lead); methodology (supporting); project administration (lead); writing – review and editing (lead).

## CONFLICT OF INTEREST STATEMENT

The authors declare that they have no conflict of interest in relation to this work.

## Supporting information


Table S1‐S2

Table S3‐S4

## Data Availability

The data in this study were deposited into Dryad with accsession links: [1] https://doi.org/10.5061/dryad.0gb5mkm7b, [2] https://doi.org/10.5061/dryad.ffbg79d1r.
