# Supplementary material for: Geographic origin shapes the adaptive divergences of Rotaria rotatoria (Rotifera, Bdelloidea) to thermal stress: Insights from ecology and transcriptomics
Source: Ecol Evol. 2024 Apr 24;14(4):e11307. doi: 10.1002/ece3.11307 (PMC11043679; doi:10.1002/ece3.11307)
Supplement: Supplementary file 1 — Table S1‐S2 [file ECE3-14-e11307-s001.docx]

**Table S1 The gene-specific primers used in this study**

| Functions | Primers | Sequences |
| --- | --- | --- |
| qRT-PCR | 18S RNA RT-F | GAAATTGTGCGCGACATCAAGGA |
|  | 18S RNA RT-R | GCAATGCCCGGGTACATGGTGGT |
|  | HSP70 RT-F | CATCAGCAGCACAGACGGAT |
|  | HSP70 RT-R | GGCATTCGAGTCATACCACCA |
|  | CAT RT-F1 | GCGGGTGAACAACCTGACTA |
|  | CAT RT-R1 | CCAACACGACGCAATGGAAA |
|  | GST RT-F | TGTCCATTCGTTCGACGTGTTA |
|  | GST RT-R | GGTCAACAGGGTGAAGACGA |
|  | Mn-SOD RT-F | TCAGCCAACAGGTGCGTTAG |
|  | Mn-SOD RT-R | GCCACAAGCTATTCGAGGTC |
|  | Cu/Zn-SOD RT-F | ACATTACATGGTCCGCGTGAA |
|  | Cu/Zn-SOD RT-R | GCCACAAGCTATTCGAGGTC |

**Table S2 Summary of sequencing data quality and the statistics of the transcriptome assemblies**

| **Sample** | **Raw Reads** | **Raw Bases** | **Valid Reads** | **Valid Bases** | **Valid%** | **Q20%** | **Q30%** | **GC%** |
| --- | --- | --- | --- | --- | --- | --- | --- | --- |
| HX_25_1 | 40454868 | 5.98G | 37188022 | 5.22G | 91.92 | 99.36 | 97.73 | 33.23 |
| HX_25_2 | 48901900 | 7.27G | 46107756 | 6.49G | 94.29 | 99.46 | 98.09 | 31.17 |
| HX_25_3 | 46599534 | 6.90G | 43215068 | 6.07G | 92.74 | 99.46 | 98.06 | 30.88 |
| HX_35_1 | 43982118 | 6.50G | 40264986 | 5.65G | 91.55 | 99.35 | 97.72 | 31.75 |
| HX_35_2 | 42872956 | 6.34G | 39332254 | 5.52G | 91.74 | 99.37 | 97.79 | 31.25 |
| HX_35_3 | 40454292 | 5.95G | 36237486 | 5.08G | 89.58 | 99.26 | 97.33 | 30.95 |
| ZJ_25_1 | 41314304 | 6.14G | 38703402 | 5.46G | 93.68 | 99.44 | 98.02 | 31.48 |
| ZJ_25_2 | 45605516 | 6.78G | 42778164 | 6.03G | 93.80 | 99.46 | 98.08 | 31.80 |
| ZJ_25_3 | 39301762 | 5.84G | 36707020 | 5.17G | 93.40 | 99.47 | 98.13 | 28.44 |
| ZJ_35_1 | 42144434 | 6.26G | 39337612 | 5.54G | 93.34 | 99.45 | 98.05 | 29.46 |
| ZJ_35_2 | 39652112 | 5.89G | 37101458 | 5.23G | 93.57 | 99.47 | 98.14 | 29.38 |
| ZJ_35_3 | 49774468 | 7.38G | 46211390 | 6.51G | 92.84 | 99.44 | 98.03 | 27.54 |

**Notes: Sample**: sample name; Raw Reads: the reads amount before filtering; **Raw Bases**: the data amount before filtering; **Valid Reads**: the valid reads amount after filtering; **Valid Bases**: the valid data amount after filtering; **Valid**: the percentage of valid reads in raw reads. **Q20**: the rate of bases which quality is greater than 20 value in clean reads; **Q30**: the rate of bases which quality is greater than 30 value in clean reads; **GC**: the percentage of G and C bases in all unigenes.
